# Facilitating Social Play for Children with PDDs: Effects of Paired Robotic Devices

**DOI:** 10.3389/fpsyg.2017.01029

**Published:** 2017-06-28

**Authors:** Soichiro Matsuda, Eleuda Nunez, Masakazu Hirokawa, Junichi Yamamoto, Kenji Suzuki

**Affiliations:** ^1^Artificial Intelligence Laboratory, Faculty of Engineering, Information and Systems, University of TsukubaTsukuba, Japan; ^2^Japan Society for the Promotion of ScienceTokyo, Japan; ^3^Department of Psychology, Keio UniversityTokyo, Japan

**Keywords:** autism spectrum disorder (ASD), social play, paired robotic devices, children, robot-mediated therapy, single subject design

## Abstract

Interacting with toys and other people is fundamental for developing social communication skills. However, children with autism spectrum disorder (ASD) are characterized by having a significant impairment in social interaction, which often leads to deficits in play skills. For this reason, methods of teaching play skills to young children with ASD have been well documented. Although previous studies have examined a variety of instructional strategies for teaching skills, few studies have evaluated the potential of using robotic devices. The purpose of the present study is to examine whether automatic feedback provided by colored lights and vibration via paired robotic devices, COLOLO, facilitates social play behaviors in children with ASD. We also explore how social play relates to social interaction. COLOLO is a system of paired spherical devices covered with soft fabric. All participants in this study were recruited as volunteers through the Department of Psychology at Keio University. The pilot study included three participants diagnosed with Pervasive Developmental Disorders (PDDs; 5- to 6-year-old boys), and compared experimental conditions with and without automatic feedback from the devices (colored lights and vibration). The results indicated that the participants in the condition that included feedback from the devices exhibited increased rates of ball contact and looking at the therapist’s ball, but did not exhibit increased rates of eye contact or positive affect. In the experimental study, a systematic replication of the pilot study was performed with three other participants diagnosed with PDDs (3- to 6-year-old boys), using an A-B-A-B design. Again, the results demonstrated that, in the condition with colored lights and vibration, the children increased ball contact as well as looking at the therapist’s ball. However, the results did not show the effect of automatic feedback consistently for three children. These findings are discussed in terms of the potential of paired robotic devices as a method to facilitate social play for children with ASD.

## Introduction

Difficulties with play skills have been well documented in children with Autism Spectrum Disorder (ASD; Wuff, 1985; Baron-Cohen, 1987; Lewis and Boucher, 1988; Jarrold et al., 1993; Charman et al., 2000; Williams et al., 2001). These difficulties are seen in sensory motor play, manipulative play, physical play, pretend play, and social play (Boucher, 1999). Consistent with this view, many studies have focused on teaching play skills to children with ASD (Jung and Sainato, 2013). Previous intervention studies have used video and live modeling (Jahr et al., 2000; MacDonald et al., 2005), pivotal response training (Stahmer, 1995; Thorp et al., 1995), activity schedules (Morrison et al., 2002; Machalicek et al., 2009), or social stories (Barry and Burlew, 2004). Researchers have also combined these strategies with contingent reinforcement (Jung and Sainato, 2013). These studies have found training increases engagement in appropriate play behavior and cooperative play in children with ASD. On the other hand, few studies have examined the effectiveness of robotic device use in teaching play skills to children with ASD, although robotic devices can automatically and immediately reinforce appropriate play behavior.

Robotic devices have been used to increase social-communication behaviors, such as joint attention (Warren et al., 2015; Simut et al., 2016) and imitation (Duquette et al., 2008), in children with ASD. These studies have focused on the use of both humanoid robots and non-humanoid toy-like robots, such as KASPER (Robins et al., 2009), Keapon (Costescu et al., 2015), NAO (Huskens et al., 2015; Warren et al., 2015), Probo (Simut et al., 2016), Robota (Billard et al., 2007), or Tito (Duquette et al., 2008). However, these robots mainly provide feedback as a result of the behavior of a child. Given that the facilitation of social play involves two people using toys, it may be necessary to consider directly providing feedback as a result of the behavior of both a child and the other individual.

Paired robotic devices might encourage cooperative behaviors, such as turn taking (Nunez et al., 2016). In this approach, remotely connected paired devices provided feedback separately as a result of child’s own behavior as well as the other individual’s behavior. Huskens et al. (2013) suggested robotic devices should be deployed as mediators to promote social interaction between a child with ASD and another individual. However, to our knowledge, no studies have examined how automatic feedback via paired robotic devices affects social play behaviors. In addition, as Diehl et al. (2012) have pointed out, most studies using robots for children with ASD have not used an experimental design, such as an experimental group design or single subject experimental design.

When considering play behaviors, we need to recognize two types. First are those related to social play, such as ball contact and looking at a therapist’s ball (Bass and Mulick, 2007). Second are those related to social interaction, such as eye contact and positive affect. The purpose of the current study is to examine whether automatic feedback via paired robotic devices facilitates social play behaviors in children with ASD, and to explore how social play relates to social interaction.

If the paired robotic devices can immediately provide automatic feedback contingent on child’s social play behaviors, it is possible that automatic feedback increases the social play behaviors in children with ASD. Therefore we hypothesized the following relationship between behavior contribution and feedback:

Hypothesis 1: The child’s ball contact and looking at the therapist’s ball will increase with automatic feedback in the form of vibration and light.

It is possible that social play will also facilitate social interaction, and then we could expect that:

Hypothesis 2a: The automatic feedback by vibration and light will increase behaviors associated with social interaction, such as eye contact and positive affect.

Alternatively, it is also possible that social play directs the child’s attention away from the therapist toward the activity, and thus we could expect that:

Hypothesis 2b: Automatic feedback in the form of vibration and light will decrease behaviors associated with social interaction, such as eye contact and positive affect.

To directly test these hypotheses a single AB design was used in a pilot study to make inferences about the effects of feedback made by colored lights and vibration via paired robotic devices on social play behaviors in three boys with PDDs. In this experiment, we used a rapidly changing reversal design with the same experimental condition as the pilot study. By using this experimental design, we further evaluated whether and what types of social play behaviors are facilitated by the feedback provided by remotely connected paired devices in children with PDDs.

## General Method

### Paired Robotic Devices: COLOLO

In the experiments, we used a system composed of paired devices, COLOLO. The devices have embedded sensors to detect when they are being manipulated, sending a message to the paired devices. This message is represented by visual cues made by colored lights and movements. Each device is made of a plastic spherical case covered by soft material. Inside there is a plate attached to the rotational axis of a motor by a microcontroller. A weight is attached to the motor and allows the sphere to wiggle by unbalancing the device. On the plate, there is a circuit board where a microcontroller, wireless communication module, tilt sensor, battery, and full color LEDs are installed. Each device is connected to a server via TCP/IP protocol. The server is a stationary computer that identifies the client device by a predefined ID. The roles of the server are to mediate communication among clients, pair/group clients, and log clients’ communication history. The microcontroller changes the color of the LEDs and sends a message to the server when the tilt sensor detects the user’s manipulation. Then, when the paired devices receive the message, the sphere starts wiggling and the color of the LEDs change according to the information in the message. In this way, users can perceive others’ actions by visualizing color changes and wiggling motions. More details on the device can be found in our previous work (Nunez et al., 2016).

### Experimental Condition

**FIGURE 1:**
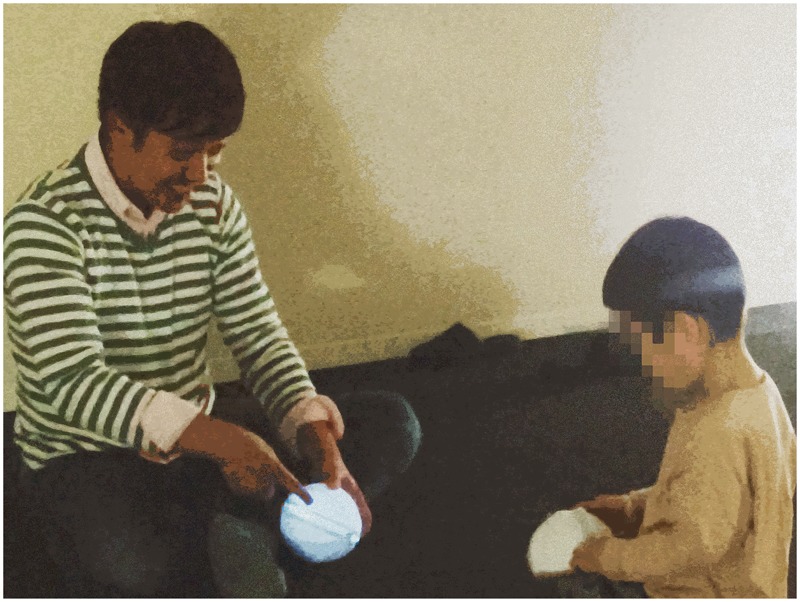
Basic image of a session in the *with* automatic feedback condition. The therapist and the child both hold COLOLO.

Both conditions (with and without automatic feedback) were implemented on the carpeted floor of a testing room at a university. In order to improve the visibility of light under the feedback condition, direct illumination was turned off and indirect lighting set at the two corners of the room (**Figure 1**). All sessions were videotaped.

**FIGURE 2:**
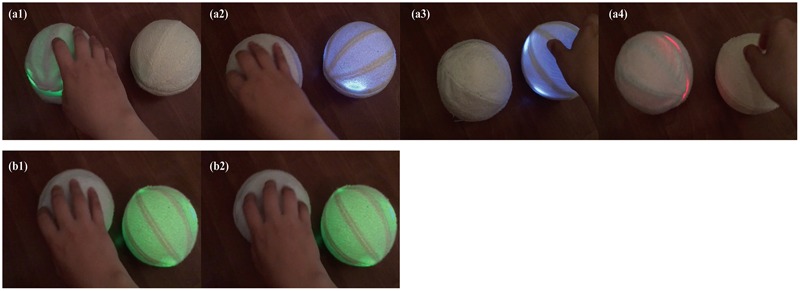
Example of the transferring lights rule. **(a1)** When a user manipulates Device A, **(a2)** the paired device (Device B) will provide feedback of light and vibration. **(a3)** When the user manipulates Device B, **(a4)** the paired device (Device A) will provide feedback of light and vibration. **(b1)** When the user manipulates Device A during the feedback of Device B, **(b2)** Device A will not give any feedback, and the user will need to wait for a response.

There were two experimental conditions. The first condition was the *with automatic feedback condition* (Phase A). The sensors embedded in the devices detected contact (e.g., handling, bouncing, or tossing) and displayed feedback using colored lights and vibration according to the interaction rule (**Figure 2**). Under this rule it is necessary to use two devices that send and receive messages triggered by the users actions (paired configuration). When the sender device is manipulated, the visual/tactile feedback is transferred to the receiver device. By doing this, the roles of the devices are switched. If a receiver device is manipulated, it will not respond to the actions until it receives the turn from the sender device. The second condition was the *without automatic feedback condition* (Phase B), in which the devices were turned off. Therefore, the child and the therapist used them as regular balls.

The study examined the differences in child social play behaviors within the two experimental conditions: with and without automatic feedback. In both conditions, the interaction took place in the following format: (1) the therapist introduced balls to the child; (2) the therapist modeled how to play with the balls (e.g., rolling, shaking, and catching them); (3) the child manipulated the balls; and (4) the child’s ball manipulating behavior was verbally/physically praised by the therapist (e.g., “You’re great!” and tickling). In addition, the therapist verbally/physically praised whenever the child made eye contact, exhibited positive affect, or approach to the therapist, throughout the session.

### Diagnosis Procedure

This study was approved by the affiliate university’s Institutional Review Board and was, therefore, completed in accordance with the ethical standards established in the 1964 Declaration of Helsinki. All participants had a diagnosis of autistic disorder, PDD-NOS, or ASD by an outside medical doctor. Diagnosis of Pervasive Developmental Disorders (PDDs) was further confirmed using the *Pervasive Developmental Disorders Autism Society Japan Rating Scale* (PARS; Kamio et al., 2006; Ito et al., 2012a). PARS, developed in Japan, is an interview-based instrument for evaluating PDDs according to DSM-IV-TR (American Psychiatric and Association, 2000). The sub and total scores of PARS have correlations with the domain and total scores of the Autism Diagnostic Interview-Revised (ADI-R; Le Couteur et al., 1989; Lord et al., 1994). All participants with PDDs met the threshold for a diagnosis of PDDs on a total peak symptom scale score (>9).

### Dependent Variables

Four dependent variables (eye contact, positive affect, ball contact, and looking at the therapist’s ball) were scored using occurrence/non-occurrence data in 15-s intervals. For each session, 20 intervals were recorded. Videotape scoring was completed by a scorer who was naïve to the purpose of the study. *Eye contact*: Eye contact was defined as the child’s looking at the therapist’s facial region. *Positive affect*: Positive affect was defined as visible and/or audible indications of happiness and enjoyment, including smiling and laughing. *Ball contact*: Ball contact was defined as the child’s contact with the ball, including handling, bouncing, and tossing the ball. *Looking at the therapist’s ball*: Looking at the therapist’s ball was scored when the child was looking at the ball that the therapist held.

### Inter-observer Agreement

Inter-observer agreements (i.e., agreements divided by agreements plus disagreements and multiplied by 100) were calculated for both the pilot study and the experimental study. The second observer was the first author, who independently scored 33% (for pilot study) and 25% (for experiment) of the sessions for four dependent variables. Agreement was calculated as the average percentage of agreement across sessions.

### Procedural Fidelity

To assess the degree to which all sessions were executed according to procedure, reliability indices for fidelity of implementation (i.e., agreements divided by agreements plus disagreements and multiplied by total number of sessions) were collected for both the pilot study and the experimental study. A research assistant and the second author completed procedural fidelity checklist on three different variables for all sessions.

## Pilot Study

### Participants

All participants were recruited as volunteers through the Department of Psychology at Keio University. Participants were three boys with PDD, “Taro,” “Sabu,” and “Jiro,” between the ages of 5 and 6 years. Names of participants have been changed to protect the participants’ identities. Informed consent was obtained from the parents before the children were included in the study.

**Table 1** displays the participants’ characteristics. The participants’ initial profiles (i.e., language, communication, motor, perceptual, and adaptive behavior skills) were assessed using standardized assessment tools: the *Kyoto Scale for Psychological Development 2001* (KSPD; Ikuzawa et al., 2002), the *Vineland Adaptive Behavior Scales*, 2nd edition Japanese version (Vineland-II; Ito et al., 2012b), and the *MacArthur Communicative Development Inventories*, Japanese version (MCDIs; Ogura, 2007). The KSPD yields standard scores for physical-movement (P-M), language-sociability (L-S), and cognitive-adaptive (C-A) subscales and total developmental quotient (DQ). The KSPD was developed for use with typically developing infants and low-function children with ASD and other developmental disorders in Japan.

**Table 1:** Participant profiles in the pilot study.

Child		Taro	Jiro	Sabu
Chronological age		6;9	5;6	5;6
PARS	Total peak symptom scale score	51	28	24
KSPD	Full DQ	77	33	38
	P-A DQ	56	56	55
	L-S DQ	76	29	34
	C-A DQ	79	45	41
VAB-II-J	Adoptive behavior composite	48	45	51
	Communication	63	34	58
	Daily living skills	47	54	61
	Socialization	38	36	45
	Motor	51	51	51
J-MCDIs	Words understood	418	74	376
	Words said	413	3	180
	Total gestures produced	37	22	35

*PARS, pervasive developmental disorders autism society Japan rating scale; KSPD, Kyoto scales of psychological development 2001; DQ, developmental quotient; Full, total scale; P-A, physical-movement; L-S, language-sociability; C-A, cognitive-adaptive; VAB-II-J, Vineland adaptive behavior scales 2nd edition Japanese version; J-MCDIs, MacArthur Communicative Development Inventories, Japanese version.*

### Design and Procedure

A single AB design was used in the pilot study. By contrasting the *with* automatic feedback condition (Phase A) and the *without* automatic feedback condition (Phase B), we could make inferences about differences of the dependent variables between the experimental conditions.

Each phase consisted of a 5-min session, and both phases were conducted in a same day for each participant. First, the *with* automatic feedback condition (Phase A) was presented, and then, after a short break, the *without* automatic feedback condition (Phase B) followed.

### Results

**FIGURE 3:**
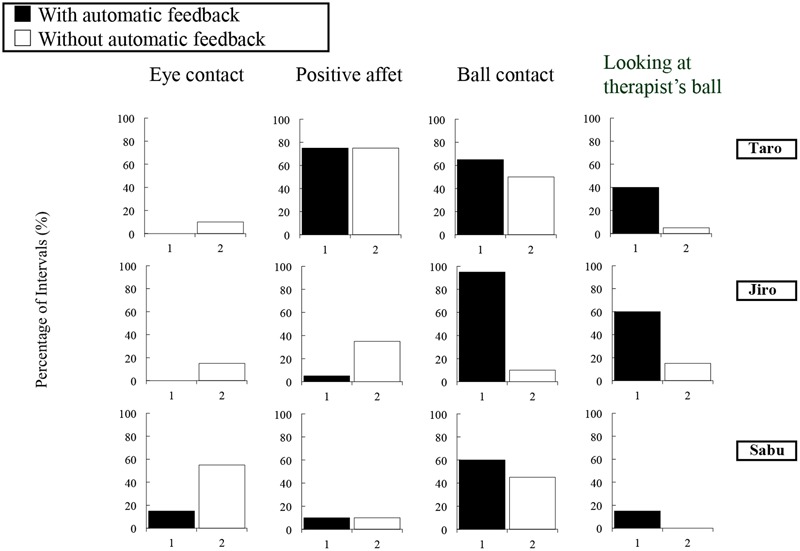
Percentage of 15-s intervals with eye contact, positive affect, ball contact, and looking at the therapist’s ball in the *with* and *without* automatic feedback conditions in the pilot study.

For eye contact, the average observer agreement value was 97% (range 95–100%); for positive affect, 97% (range 95–100%); for ball contact, 90% (range 85–95%); and for looking at the therapist’s ball, 82% (range 80–85%). Fidelity of implementation for socially/physically reinforcing the child’s eye contact, positive affect, and approach to the therapist averaged 67%; fidelity of implementation for socially/physically reinforcing the child’s ball contact averaged 100%; and fidelity of implementation for modeling and prompting ball play averaged 100%. Results are shown in **Figure 3**.

#### Eye Contact

The percentage of intervals with eye contact in the *with* automatic feedback condition was 0% for Taro, 0% for Jiro, and 15% for Sabu. On the other hand, in the *without* automatic feedback condition, these numbers increased to 10, 15, and 55%, respectively.

#### Positive Affect

Taro and Sabu demonstrated almost the same levels of positive affect in both conditions. Jiro exhibited positive affect in 5% of the intervals in the *with* automatic feedback condition and 35% of the intervals in the *without* automatic feedback condition.

#### Ball Contact

All three children demonstrated increased levels of ball contact in the *with* automatic feedback condition. Specifically, the percentage of intervals with ball contact in the *with* automatic feedback condition was 65% for Taro, 95% for Jiro, and 60% for Sabu. In contrast, in the *without* automatic feedback condition, these figures decreased to 50, 10, and 45%, respectively.

#### Looking at the Therapist’s Ball

Similarly, all three children exhibited increased levels of looking at the therapist’s ball in the *with* automatic feedback condition. Specifically, the percentage of intervals with looking at the therapist’s ball during the *with* automatic feedback condition was 40% for Taro, 60% for Jiro, and 15% for Sabu. In contrast, during the *without* automatic feedback condition, these numbers decreased to 5, 15, and 0%, respectively.

## Experimental Study

### Participants

All participants were recruited as volunteers through the Department of Psychology at Keio University. The participants were three boys with ASD, “Shiro,” “Goro,” and “Riku,” between the ages of 3 and 6 years. Names of participants have been changed to protect the participants’ identities. Informed consent was obtained from the parents before the children were included in the study. **Table 2** displays the participants’ characteristics.

**Table 2:** Participant profiles in the experiment.

Child		Shiro	Goro	Riku
Chronological age		5;8	6;8	3;8
PARS	Total peak symptom scale score	52	46	28
KSPD	Full DQ	45	74	44
	P-A DQ	54	57	65
	L-S DQ	41	68	25
	C-A DQ	49	79	45
VAB-II-J	Adoptive behavior composite	55	53	49
	Communication	51	68	43
	Daily living skills	60	45	56
	Socialization	69	45	41
	Motor	51	67	65
J-MCDIs	Words understood	199	418	47
	Words said	181	338	11
	Total gestures produced	52	51	25

*PARS: pervasive developmental disorders autism society Japan rating scale; KSPD, Kyoto scales of psychological development 2001; DQ, developmental quotient; Full, total scale; P-A, physical-movement; L-S, language-sociability; C-A, cognitive-adaptive; VAB-II-J, Vineland adaptive behavior scales 2nd edition Japanese version; J-MCDIs, MacArthur Communicative Development Inventories, Japanese version.*

### Design and Procedure

The Council for Exceptional Children (CEC) Division of Research established a task force to develop guidelines for evidence-based practices (Odom et al., 2004). The task force identified four types of research methodologies: qualitative, correlational, experimental group, and single subject designs (Odom et al., 2004). Single subject designs have been used to compare the causal relationship between independent and dependent variables (Barlow et al., 2009). In this experiment, we used a single subject experimental design in a particular, rapidly changing reversal design (Cooper et al., 1990, 1993; Dunlap et al., 1991; Ishizuka and Yamamoto, 2016) over a total of two experimental days to compare the effects of lighting and vibration as automatic feedback. For all children, the experiment consisted of four 5-min sessions. Each participant had two 5-min sessions per day.

### Results

**FIGURE 4:**
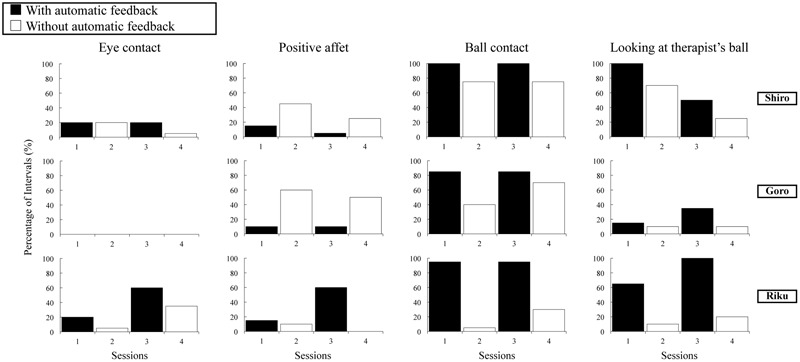
Percentage of 15-s intervals with eye contact, positive affect, ball contact, and looking at the therapist’s ball in the *with* and *without* automatic feedback conditions in the experimental study.

For eye contact, the average observer agreement value was 80% (range 75–85%); for positive affect, 88% (range 85–90%); for ball contact, 95% (range 85–100%); and for looking at the therapist’s ball, 88% (range 75–100%). Fidelity of implementation for socially/physically reinforcing the child’s eye contact, positive affect, and approach to the therapist averaged 92%; fidelity of implementation for socially/physically reinforcing the child’s ball contact averaged 92%; and fidelity of implementation for modeling and prompting ball play averaged 100%. Results of the reversal analyses for each of the dependent variables are presented in **Figure 4**.

#### Eye Contact

Shiro exhibited eye contact with a mean of 20% of the intervals in the *with* automatic feedback condition and a mean of 12.5% in the *without* automatic feedback condition. Goro showed no eye contact in either condition. In the *with* automatic feedback condition, Riku exhibited eye contact for a mean of 40% of the intervals. On the other hand, in the *without* automatic feedback condition, his eye contact decreased to a mean of 20% across sessions.

#### Positive Affect

Shiro and Goro demonstrated a similar response pattern for positive affect. Specifically, in the initial *with* automatic feedback probe, they exhibited low positive affect. With the introduction of the *without* automatic feedback condition, their levels of positive affect increased to 45% (for Shiro) and 60% (for Goro) of the intervals. The reintroduction of the *with* automatic feedback condition was accompanied by a drop in positive affect levels to 5% and 10% of the intervals, respectively. The final *without* automatic feedback condition phase resulted in positive affect for 25 and 50% of the intervals, respectively, for the two boys.

In the first *with* automatic feedback probe, Riku exhibited positive affect in 15% of the intervals. Following the introduction of the *without* automatic feedback condition, his positive affect decreased slightly to 10% of the intervals. During the reintroduction of the *with* automatic feedback condition, Riku exhibited positive affect in 60% of the intervals. In the final *without* automatic feedback condition phase, Riku did not exhibit any positive affect.

#### Ball Contact

All three children demonstrated similar response patterns for ball contact. The initial *with* automatic feedback phase resulted in ball contact in 100% (for Shiro), 85% (for Goro), and 95% (for Riku) of the intervals. With the introduction of the *without* automatic feedback condition, the levels of ball contact decreased to 75%, 40%, and 5%, respectively. The reintroduction of the *with* automatic feedback condition was accompanied by a rise in ball contact levels to 100, 85, and 95% of the intervals, respectively. The final *without* automatic feedback condition phase resulted in ball contact for 75, 70, and 30% of the intervals, respectively, for the three boys.

#### Looking at the Therapist’s Ball

Shiro exhibited looking at the therapist’s ball with a mean of 75% in the *with* automatic feedback condition and a mean of 47.5% in the *without* automatic feedback condition. For Goro, the means were 25% in the *with* automatic feedback condition and 10% in the *without* automatic feedback condition. In the *with* automatic feedback condition, Riku exhibited looking at the therapist’s ball for a mean of 82.5% of the intervals. In contrast, during the *without* automatic feedback condition, his looking at the therapist’s ball decreased to a mean of 15% across sessions.

## General Discussion

This study investigated the effects of automatic feedback in the form of colored lights and vibration produced via paired robotic devices, COLOLO, in social play and interaction in children with ASD. The frequency of ball contact and looking at the therapist’s ball were higher in the *with* automatic feedback condition than in the *without* automatic feedback condition, supporting Hypothesis 1. On the other hand, the frequencies of eye contact and positive affect for all children with ASD did not consistently increase or decrease in the *with* automatic feedback condition, thus the results indicated lack of support for both Hypothesis 2a and Hypothesis 2b. Therefore, when using the paired robotic devices, the children with ASD appear to have exhibited increases in social play behaviors using toys and but not increases in behaviors associated with social interaction.

Considering ball contact, Hypothesis 1 was positively supported. The findings are in lines with one of the pioneering works, which has demonstrated that a spherical mobile robot, Roball, may increase a child’s interaction with a ball by providing automatic feedback consisting of motion, messages, sounds, and an illuminating interface (Michaud et al., 2005). This suggests that automatic feedback of vibration (tactile stimulus) might function as a reinforcer for ball contact behavior. However, we also used light feedback (visual stimulus). There is a possibility that light feedback also functions as a reinforcer for child’s ball contact. Therefore, in a future study, we would evaluate which modality of feedback has a stronger effect on increasing ball contact.

Considering frequency of looking at the therapist’s ball, the first Hypothesis was also positively supported. This indicates that attention to shared play materials might be increased by light feedback via paired robotic devices. Although we used vibration feedback, this feedback was not contingent upon the child looking at the therapist’s ball, but contingent on the child looking at his own ball. Thus, light feedback provided via remotely connected paired devices may increase attention to the play materials of peers in children with ASD.

Concerning Hypotheses 2a and 2b of the current study, our results did not support either of these hypotheses. Neither the child’s eye contact nor their positive affect consistently increased as a result of the feedback in the form of light and vibration. The result can be easily interpreted because the feedback was not contingent upon the child’s responses. In addition, however, increases in eye contact and positive affect were observed in the *withou*t automatic feedback condition for two children. A potential explanation for this outcome could be the frequency of the reinforcement provided by the therapist. As far as ecologically validity is concerned, in the procedure of this experiment, the therapist provided verbal/physical praise for the child’s eye contact and positive affect throughout the session. This may have led to increased opportunity for praise for the therapist in the *withou*t automatic feedback condition in which the frequencies of child’s ball contact was lower. To improve this aspect of the intervention, we recommended that future studies include the combined use of other wearable devices, such as an eye tracker (Ye et al., 2012) or a face reader device we have developed for detecting smiles from facial EMG signals (Gruebler and Suzuki, 2014), in order to provide contingent feedback for child eye contact and/or positive affect.

There were several limitations to the current study. First, we used a single subject experimental design with three children with ASD in this study, and this limits the generalizability of the results to the larger population due to limitations inherent in single subject experimental designs, such as absence of statistical analysis and inference. Further studies are required, including use of a group experimental design with larger sample sizes. Second, although we used an ABAB design to minimize carryover effects, because the experiment sessions were administered across 2 days, we were not able to eliminate ordering and novelty effects. It is possible that the novelty of the interaction affected the increase in the dependent variables on the 1st day (first set of AB trials) and on the 2nd day (second set of AB trials), due to the time that has elapsed between the first and the second session. Further studies must seek to eliminate ordering and novelty effects through blocked and longitudinal study designs. Third, we need to be cautious about interpreting the observed increases in children’s ball contact and looking at the therapist’s ball as the result of automatic feedback functioning as a reinforcer. It was unclear whether automatic feedback functioned as an antecedent stimulus or a reinforcer for children’s ball contact and looking at the therapist’s ball. Further research is warranted to identify the function of automatic feedback via the implementation of a yoked condition. Fourth, we only used the feedback of light (visual stimulus) and vibration (tactile stimulus). Future studies will be required to use other modalities, such as sound (auditory stimulus).

Nevertheless, the current findings establish that feedback via paired robotic devices can facilitate some aspects of social play behaviors in children with ASD, whereas previous studies have focused on examining differences between a human and a robot as an interaction partner (e.g., Costescu et al., 2015; Srinivasan et al., 2015; Simut et al., 2016), or investigating the effects of teaching by the robot (e.g., Billard et al., 2007; Warren et al., 2015). As Huskens et al. (2013) have suggested, it would be interesting to see more studies on this topic; in other words, there is a wide range of necessities for further investigation. While we are hopeful that clinical applications of paired robotic devices may demonstrate significant enhancement of social play for children with ASD at an early developmental stage, it is important to note that future research should reveal both whether and how the paired robotic devices contribute to increasing various forms of social play behaviors in children with ASD.

## Ethics Statement

This study was carried out in accordance with the recommendations of Keio University’s Institutional Review Board with written informed consent from all parents of participants.

## Author Contributions

SM, EN, MH, JY, and KS designed the research. SM and EN performed the research. SM analyzed the data and wrote the article.

## Conflict of Interest Statement

The authors declare that the research was conducted in the absence of any commercial or financial relationships that could be construed as a potential conflict of interest.
